# Growing teratoma syndrome of the ovary: a case report and literature review

**DOI:** 10.3389/fonc.2024.1412206

**Published:** 2024-07-15

**Authors:** Jiaying Tao, Zhixian Shi, Mulan Li, Tingting Li

**Affiliations:** ^1^ Department of Obstetrics and Gynecology, Chengdu Women’s and Children’s Central Hospital, School of Medicine, University of Electronic Science and Technology of China, Chengdu, China; ^2^ West China Second University Hospital, Sichuan University, Chengdu, China

**Keywords:** growing teratoma syndrome (GTS), ovarian immature teratoma, ovarian cancer, chemoradiotherapy resistance, early diagnosis

## Abstract

Growing teratoma syndrome (GTS) is a rare condition that arises secondary to malignant germ cell tumors. It is characterized by an enlarging abdominal mass during or after chemotherapy, normal tumor markers, and histopathological indications of mature teratoma components. Awareness of GTS is limited, and it is often mistaken for disease progression or recurrence. This misdiagnosis can lead to delayed treatment and increased risk of complications. Therefore, early identification of GTS is crucial to avoid unnecessary systemic treatments and reduce financial burden. GTS is unresponsive to chemotherapy or radiotherapy and complete surgical resection is the sole therapeutic strategy. In this report, we present a case of GTS in a 20-year-old female following treatment for immature teratoma, alongside a review of the relevant literature aimed at enriching our insight into the clinical manifestations of GTS.

## 1 Introduction

Ovarian immature teratoma (IMT) is one of the most common histological subtypes of malignant ovarian germ cell tumors, comprising tissues from all three germ layers as well as immature neural elements, and accounting for approximately one-third of cases Smith et al. (1). IMT predominantly occurs in young women, and given its incidence at a relatively young age, the primary treatment approach focuses on preserving fertility. This is typically achieved through fertility-sparing unilateral salpingo-oophorectomy, followed by adjuvant chemotherapy with the BEP (Bleomycin, Etoposide, and Cisplatin) regimen.

Growing teratoma syndrome (GTS) represents an extremely rare metastatic complication arising from malignant germ cell tumors Amsalem et al. (2). The phenomenon was first delineated by DiSAIA et al. (3), who observed a ‘chemotherapy-induced transformation’ in three female patients with ovarian immature teratoma, where post-chemotherapy, immature tumor components evolved into mature elements. Subsequently, Logothetis et al. (4) reported six cases of testicular malignant germ cell tumors that recurred as mature teratomas following successful chemotherapy and coined the term GTS to describe these occurrences. Specifically, GTS is defined by three specific criteria: 1) Continuously enlarging abdominal mass during or after chemotherapy; 2) Previously elevated serum tumor markers are now within normal limits; 3) Pathological examination of the resected tumor reveals only mature teratoma components Logothetis et al. (4). In 2004, Amsalem et al.’s research suggested that ‘chemotherapy-induced transformation’ and ‘GTS’ appear to be the same phenomenon Amsalem et al. (2). Here, we present a case of a 20-year-old female with ovarian immature teratoma who developed GTS following treatment. Additionally, we conducted a retrospective literature review to enhance our understanding of this unique syndrome.

## 2 Case presentation

**Figure 1:**
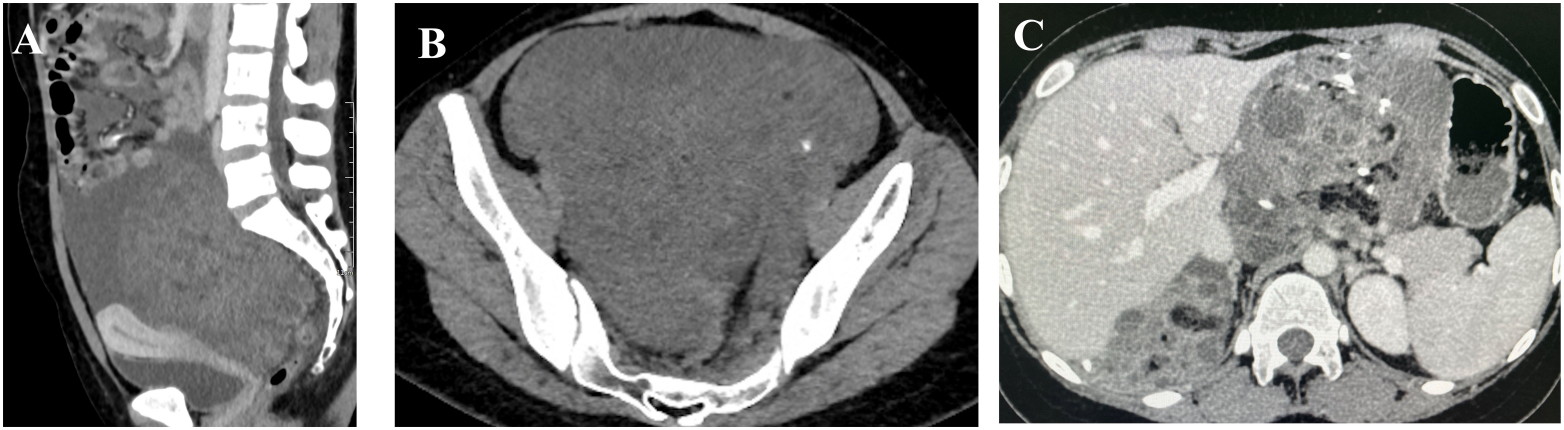
Images **(A, B)** (taken on 2022–12-14) reveal a large solid-cystic mass occupying the lower abdomen and pelvic cavity, which contained scattered punctate calcifications and shadows with fat density; image **(C)** (taken on 2023–11-27) demonstrates multiple masses in the pelvic and abdominal cavities fused into a huge irregular continuous mass.

**Figure 2:**
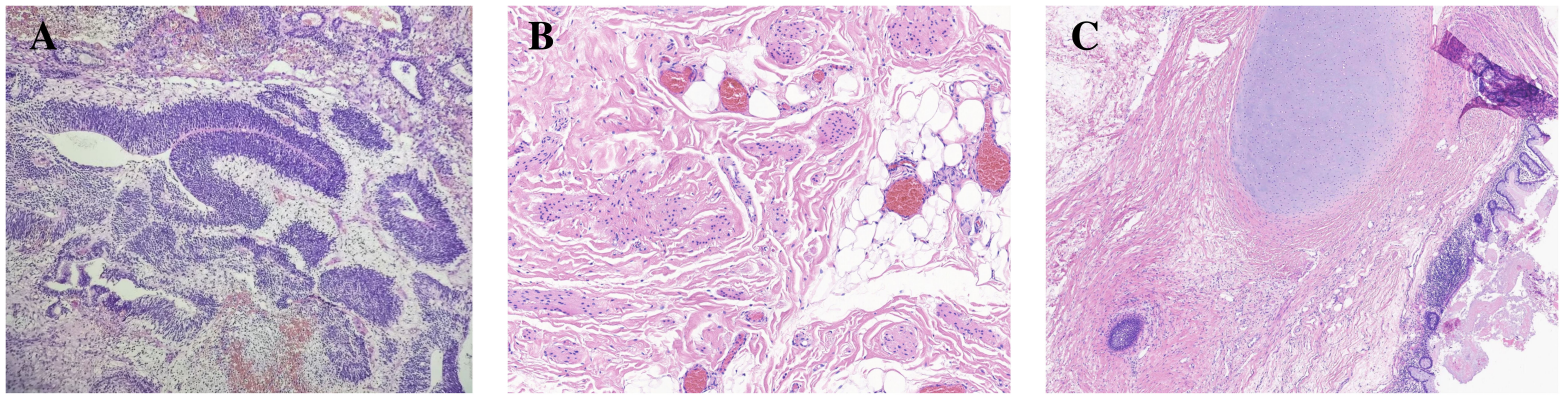
Representative histology of immature and mature teratoma. In image **(A)** (HE x100), the resected specimen of original ovarian immature teratoma showing primitive neural tube. Mature teratoma after chemotherapy in the same patient composed of various tissue components such as fat and cartilage (image **B**, HE x100), as well as neuroglia and intestinal epithelium (image **C**, HE x40).

In December 2022, a 20-year-old woman presented to a local hospital with symptoms of abdominal pain and distension lasting for 10 days. An ultrasound examination revealed a solid-cystic mixed echoic mass in the pelvic cavity, with indistinct borders and irregular morphology. Multiple small hypoechoic areas were observed within the mass, and abundant blood flow signals were detected. Additionally, a fluid-filled dark area was visible in both the abdominal and pelvic cavity. She was then admitted to Chengdu Women’s and Children’s Central Hospital. Computed Tomography (CT) scans revealed a huge solid-cystic mass occupying the lower abdomen and pelvic cavity where the mass contained scattered punctate calcifications and fat density shadows. Multiple nodular shadows were observed in the peritoneum and greater omentum (**Figures 1A, B**). Serum tumor markers were elevated: *α*-fetoprotein (AFP) = 833.1*ng/ml*, *CA*125 = 422.1*U/ml*, *CA*19−9 = 81.81*U/ml*, *β*-human chorionic gonadotropin (HCG) was negative.During the surgical exploration, a significant amount of yellow ascites was observed. An irregular mass was found attached to the left ovary, and multiple nodules were observed in the omentum. No obvious masses were observed on either ovary or on the peritoneal surfaces, and no enlarged retroperitoneal lymph nodes were palpable. The mass and a portion of the omentum were resected, and random peritoneal biopsies were taken from the pelvis, paracolic gutters, and undersurfaces of the diaphragm. The mass was completely resected without rupture, and there was no residual gross lesion after the operation. Cytological examination of the ascites did not detect malignant cells. Histopathological analysis of the mass and omentum revealed a stage IIIc high-grade immature teratoma (grade 3), with abundant immature intestinal epithelium present within the tumor, and no distinct yolk sac tumor components were identified (**Figure 2A**). The patient was then administered 3 cycles of BEP (bleomycin, etoposide, cisplatin, bleomycin 30 units IV per week plus etoposide 100 mg/m2 IV daily on days 1–5 plus cisplatin 20 mg/m2 IV daily on days 1–5, repeated every 21 days) as adjuvant chemotherapy. After 3 cycles of chemotherapy, the tumor marker (AFP) returned to normal, and an MRI showed no masses in the pelvic or abdominal cavity. She achieved a complete clinical response and was recommended for regular follow-up.

**Figure 3:**
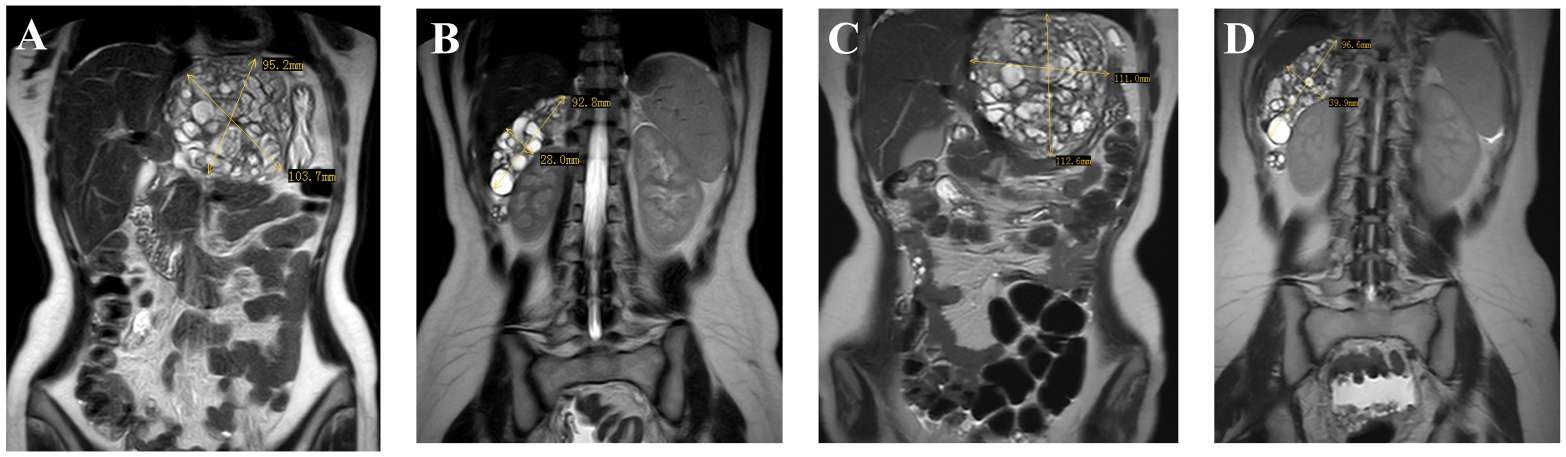
Images **(A, B)** (taken on 2023–07-19) indicate multiple masses in the pelvic and abdominal, with the largest located in the hepatogastric space; images **(C, D)** (taken on 2023–09-24) show an enlargement of the pelvic and abdominal masses.

In May 2023, three months after the last cycle of chemotherapy, the patient was followed up at a local hospital where a positron emission tomography (PET)/CT scan revealed an abdominal mass measuring approximately 10 cm, with normal tumor markers. She was then admitted to West China Second University Hospital where she received two cycles of BEP regimen chemotherapy. Despite this, an abdominal MRI examination showed no significant reduction in the size of the mass (**Figures 3A, B**). Given the limited effectiveness of chemotherapy, the patient was subsequently administered 3 cycles of chemotherapy with ‘paclitaxel, ifosfamide, and cisplatin’. However, the mass continued to grow during this chemotherapy (**Figures 3C, D**). Consequently, the patient was admitted to The West China University Hospital for surgery. Preoperative CT results showed multiple masses in the pelvic and abdominal cavities fused into a huge irregular continuous mass (**Figure 1C**). Intraoperatively, a huge cystic-solid mass measuring approximately 20x15x13 cm was found deep in the left upper abdomen, behind the left lobe of the liver and above the lesser curvature of the stomach, along with multiple cystic-solid masses fused in the right liver posterior space and diaphragm, measuring approximately 6x5x7 cm. Multiple cystic-solid masses were also found in the pelvic peritoneum, as well as multiple nodules in the mesentery and greater omentum ranging in size from 0.2 cm to 2 cm. A fertility-preserving surgery was conducted, which involved the resection of the posterior peritoneal tumor located behind the left lobe of the liver, the diaphragm tumor, and the greater omentum, along with the excision of the pelvic tumor. Postoperatively, no macroscopic residual lesions were observed. Postoperative pathological examination revealed various differentiated tissues, including cartilage, neural tissue, glandular and squamous epithelium, sebaceous glands, along with keratinization, necrosis, and foam cell accumulation. No immature components were detected (**Figures 2B, C**). Based on the clinical progression of an enlarging abdominal mass post-chemotherapy, normalized serum tumor markers, and pathological evidence of mature teratoma, the patient was diagnosed with ‘ovarian GTS’. Following NCCN guidelines Armstrong et al. (5), we outline the follow-up plan for this patient: 1) In the first year, perform physical exams and serum tumor marker tests every 2 months, with chest/abdominal/pelvic (C/A/P) CT scans every 3–4 months. 2) In the second year, continue with physical exams and serum tumor marker tests every 2 months, and C/A/P CT scans every 4–6 months. 3) During the third year, continue with physical exams and serum tumor marker tests every 4–6 months, and A/P CT scans every 6–12 months. 4) In the fourth and fifth years, continue with physical exams and serum tumor marker tests every 6 months, and A/P CT scans every 6–12 months. 5) After five years, continue with annual physical exams and serum tumor marker tests, and perform CT scans as needed based on clinical symptoms. Three months after surgery for GTS, this patient is alive with no evidence of disease.

## 3 Discussion

GTS manifests with an incidence of 1.9%–7.6% in male non-seminomatous germ cell tumors of the testis Lee et al. (6). In females, its prevalence is rarer and the exact incidence is somewhat nebulous, though some reports suggest a rate of approximately 12% Zagamé et al. (7). For patients with immature teratomas, the potential progression to GTS is estimated around ∼20% Wang et al. (8). A review of 101 cases of ovarian GTS found that GTS mostly occurs in adolescents and young adults, with a median onset age of 22 years at the time of primary diagnosis of immature teratoma Li et al. (9).

The etiology of GTS remains elusive, with two prevailing theories cited in the literature to elucidate its pathogenesis. 1) Chemotherapy eliminates the chemotherapy-sensitive immature components, while chemotherapy-insensitive mature components continue to grow, leading to GTS as the disease progresses André et al. (10). 2) Chemotherapy alters cellular dynamics, causing pluripotent malignant germ cells to transform into mature teratomas, acquiring a benign phenotype insensitive to chemotherapy, and proliferating autonomously DiSAIA et al. (3). Additionally, Hong et al. (11) have proposed a hypothesis suggesting that malignant cells may inherently or spontaneously differentiate into benign tissue, with this process potentially being extended by therapeutic intervention as part of the natural progression.

The risk factors that precipitate GTS remain unclear. In male patients, mature teratoma components within the primary tumor has been identified as a predictive factor for GTS André et al. (10). Research indicates that residual disease post-initial surgery and peritoneal gliomatosis are independent risk factors for the occurrence of GTS Wang et al. (8). Additionally, André et al. (10) suggested that the presence of mature teratoma components within the primary tumor, inadequate initial surgery, and metastatic disease unresponsive to chemotherapy contribute to an increased risk of GTS. Moreover, Tangjitgamol et al. (12) showed that tumor rupture during surgery might be associated with the occurrence of GTS.

The retroperitoneum is recognized as the most frequent locus for the occurrence of GTS. Additional sites of manifestation have been documented, encompassing the lungs, neck, supraclavicular region, inguinal lymph nodes, mediastinum, forearm, mesentery, and liver Zagamé et al. (7). Generally, most GTS nodules following ovarian germ cell tumors are localized to the pelvis, abdomen, and retroperitoneum, rather than exhibiting distant systemic spread Wang et al. (8) Djordjevic et al. (13). However, the exact mechanisms of disease dissemination in GTS are not fully understood. Research by Shibata et al. (14) highlighted a case of ovarian GTS that demonstrated three concurrent pathways of metastatic spread: direct extension, lymphatic dissemination, and hematogenous routes. This diversity in potential spread underscores the complex behavior of GTS and highlights the need for further investigation to better understand its pathophysiology.

Diagnosing GTS requires a collaborative approach involving the patient’s medical history, treatment details, and coordination among gynecology, ultrasonography, radiology, and pathology departments. GTS is often misidentified as either disease progression or recurrence. If pelvic or abdominal growth of masses is found during or after the chemotherapy, the IOTA ADNEX model and tumor marker levels can help diagnosis to some extent. Elevated tumor markers generally signify a recurrence of IMT, whereas GTS tends to correspond with normal or slightly elevated tumor marker levels (only a few studies have noted slight elevation in tumor markers) Lorusso et al. (15). Ultrasound assessment by an expert, or the use of the IOTA ADNEX model in conjunction with the tumor marker profiles, can often indicate the specific subtype of malignancy Timmerman et al. (16). However, the clinical behaviors of recurrence in IMT (true recurrence and mature recurrence) are not fully understood. Surgery should be performed to evaluate the nature of the relapse, determining whether it is an IMT requiring further adjuvant chemotherapy or mature elements needing no further management Wang et al. (17).

The clinical manifestations of GTS are related to the location of tumor growth, which can lead to compression of surrounding organs and evoke an array of clinical symptoms such as pain, intestinal obstruction, renal failure due to ureteral compression, thrombophlebitis, and tissue necrosis. According to Li et al. (9), the median size of the primary tumor at diagnosis was reported to be 18.7 cm (range: 6−45 cm), median subsequent tumor size was 8.6 cm (range: 1−25 cm). The median tumor growth rate during the interval between primary treatment and the diagnosis of ovarian GTS was 0.94 cm/month (range: 0.3−4.3 cm/month). Furthermore, the median duration leading to the diagnosis of GTS post-treatment was 26.6 months (range: 1−264 months).

The imaging diagnosis of GTS predominantly relies on CT scans. The maturation characteristics discernible via CT include an increased density within the mass, well-delineated margins, and the emergence of internal calcifications, alongside the presence of fatty deposits and cystic alterations Moskovic et al. (18). PET/CT scans are regarded as furnishing more comprehensive diagnostic insights than CT alone, owing to their enhanced capability to differentiate between active disease and benign processes, thereby offering a more detailed evaluation of the syndrome Kikawa et al. (19).

GTS is known for its resistance to both chemotherapy and radiotherapy, rendering surgical intervention the sole method of treatment André et al. (10). According to the second pathogenic mechanism, if GTS occurs as a result of chemotherapy reversal, it seems logical that it retains a high level of histological type and malignant potential. Complete surgical resection achieving R0 status significantly improves prognosis, with a 5-year survival rate of 89%−90% Wang et al. (8). The risk of recurrence after complete resection is very low, ranging from 0%*to*4%. In contrast, the recurrence rates post-partial resection are notably higher, ranging from 72% to 83% Spiess et al. (20).

However, surgery is challenging and often involves partial organ resection and reconstruction procedures. Despite these challenges, complete resection is advocated even when vital organs are involved. Given that GTS tumors frequently involve multiple organs and multiple metastases, the final management should be determined within a multidisciplinary team or experienced centers, taking into account both the diagnostic findings and the overall patient profile Timmerman et al. (16) Bentivegna et al. (21)Pashankar et al. (22). In scenarios where surgery is unfeasible or complete resection cannot be accomplished, alternative treatments such as Pazopanib have been reported in the literature as therapeutic options for GTS Schultz et al. (23). The overall prognosis for patients with GTS is generally positive.

GTS has a risk of malignant transformation, where the teratoma may evolve into more aggressive forms of cancer such as sarcoma, squamous cell carcinoma, adenocarcinoma, or adenosquamous carcinoma. The incidence rate of such malignant transformations is reported to be between 3% and 5.4% André et al. (10) Shigeta et al. (24). Considering this risk, it is crucial to maintain regular follow-up through imaging studies and serum tumor marker evaluations to promptly detect any signs of malignant change, ensuring timely intervention and management.

Ovarian GTS predominantly occurs in young women, spotlighting fertility preservation as a critical concern. For patients with stage II to IV immature teratomas, postoperative chemotherapy is recommended, though it may permanently impair reproductive functions. Cryopreservation of ovarian tissue is the primary option to preserve fertility. Fertility-sparing surgery should be considered for those who wish to retain their fertility Perelli et al. (25, 26). In an extensive review of 101 cases involving patients with ovarian GTS, Li et al. (9) reported that 5 patients conceived in the period between the primary diagnosis and the diagnosis of GTS, with 1 patient achieving pregnancy post-diagnosis of ovarian GTS. In the study by Bentivegna et al, among 38 patients with Ovarian GTS, 20 underwent fertility-sparing surgery, of which 4 out of 6 patients who planned to conceive became pregnant naturally, and 1 successfully conceived using assisted reproductive technology Bentivegna et al. (21). These findings underscore the feasibility and significance of fertility-sparing surgical approaches in managing Ovarian GTS, with both studies advocating for such interventions ‘when possible’. However, the ambiguity surrounding the criteria to determine when fertility-sparing surgery is feasible highlights a gap in the existing literature. This lack of clarity calls for additional research to establish definitive guidelines that can aid in making informed decisions about preserving fertility in patients with Ovarian GTS.

## 4 Conclusion

GTS is a rare condition. When an abdominal mass enlarges during or after chemotherapy for immature teratoma, with normal serum tumor markers, GTS should be considered. A definitive diagnosis typically hinges on pathological examination after surgical resection. Complete surgical resection is crucial for a good prognosis and is essential in GTS management. Given the potential for malignancy, stringent and continuous follow-up is essential to monitor for any signs of progression or transformation.

## Data availability statement

The raw data supporting the conclusions of this article will be made available by the authors, without undue reservation.

## Ethics statement

The studies involving humans were approved by Ethics Committee of Chengdu Women and Children’s Central Hospital. The studies were conducted in accordance with the local legislation and institutional requirements. The participants provided their written informed consent to participate in this study. Written informed consent was obtained from the individual(s) for the publication of any potentially identifiable images or data included in this article.

## Author contributions

JT: Writing – original draft, Conceptualization. ZS: Data curation, Writing – review & editing. ML: Investigation, Writing – review & editing. TL: Validation, Writing – review & editing.
